# Tongue hyperpigmentation associated with chemotherapy

**DOI:** 10.3402/jchimp.v3i3-4.21047

**Published:** 2013-12-17

**Authors:** Majd Alfreijat

**Affiliations:** Department of Medicine, MedStar Union Memorial Hospital, Baltimore, MD, USA

**Keywords:** hyperpigmentation, drug reaction, chemotherapy, therapies

We demonstrate a new hyperpigmentation on the tongue of a 68-year-old female patient who was diagnosed with stage IIA infiltrating ductal carcinoma of the left breast who underwent modified radical mastectomy followed by a triple chemotherapy regimen which consisted of doxorubicin, docetaxel, and cyclophosphamide.

The patient developed the tongue hyperpigmentation a few weeks after the initiation of chemotherapy. She never had similar lesions in the past, nor had any involvement of other parts of the body, including palms, soles, trunk, nails, or scalp. A few months after stopping the chemotherapy, the patient had a remarkable improvement in her tongue discoloration.

## Case discussion

Hyperpigmenation of the tongue following a treatment with chemotherapy has been previously reported (1–3). Both doxorubicin and cyclophosphamide have been noted to be the single culprit agent in several cases (4), whereas docetaxel has been associated with dark pigmentation of the nail (5).

The exact pathophysiology is still not well understood; it has been suggested that melanocyte-stimulating hormone (MSH) may be boosted by certain chemotherapy agents like doxorubicin (6), and that this may account for the higher prevalence of this side effect in dark-skinned patients.

**Figure:**
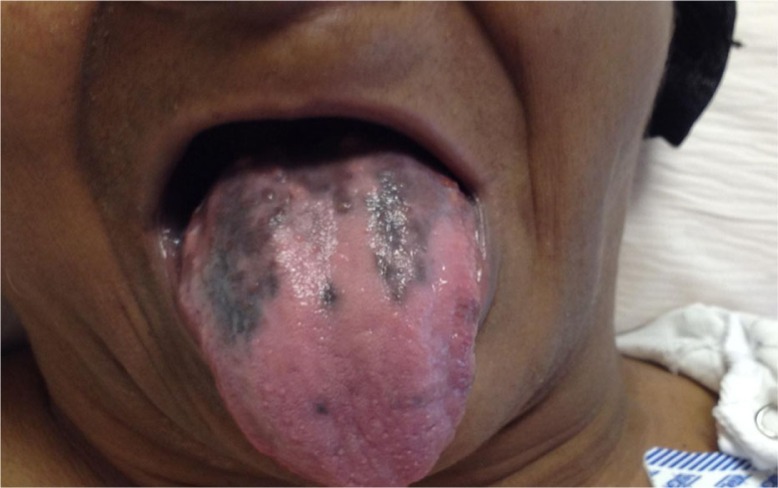


It is usually self-limited and disappears a few weeks after treatment is completed.
